# Dementia in Latin America: An Emergent Silent Tsunami

**DOI:** 10.3389/fnagi.2016.00253

**Published:** 2016-10-28

**Authors:** Sandra Baez, Agustín Ibáñez

**Affiliations:** ^1^Laboratory of Experimental Psychology and Neuroscience, Institute of Cognitive and Translational Neuroscience, INECO Foundation, Favaloro UniversityBuenos Aires, Argentina; ^2^Departamento de Psicología, Universidad de los AndesBogotá, Colombia; ^3^Departamento de Psicología, Universidad Autónoma del CaribeBarranquilla, Colombia; ^4^Center for Social and Cognitive Neuroscience, School of Psychology, Universidad Adolfo IbáñezSantiago de Chile, Chile; ^5^Australian Research Council Centre of Excellence in Cognition and its DisordersSydney, NSW, Australia

**Keywords:** Latin America, dementia, priority, barriers, diagnosis

Recently the Lancet Neurology Commission (Winblad et al., 2016) has provided expert recommendations and highlighted that European Union (EU) is well positioned to take the work lead to prevent and cure the Alzheimer's disease and other dementias, and to provide models for care. This panorama strongly contrasts with the one of Latin America. Although there is an evident growing interest in dementia among Latin American countries (LAC) (Lancet, 2015), important barriers in this region involves big challenges to join the fight against dementia. In this article, we identify some key issues regarding dementia diagnosis that could trigger immediate actions in LAC, contrasting them with the EU scenario (Winblad et al., 2016).

Demographic characteristics of LAC have substantially changed over the past 25 years, with an extensive decline of mortality and life expectancy increasing (Barreto et al., 2012). Demographical transitions have contributed to a large and rapid growth in the number of people suffering from dementia (Sousa et al., 2010). Predictions suggest that by 2050, the number of people aged 60 years will increase by 1.25 billion, with 79% living in the world's less developed regions (Prince et al., 2013). In spite of the huge economic and social impact that dementia is causing in LAC (Manes, 2016), loss of awareness and deficiencies in health system are more accentuated in LAC than in the EU. Some of these obstacles are addressed in this article, including the limited access to health facilities, the need for standardizing diagnostic practices, and the existing barriers regarding resources and culture.

In LAC, the diagnosis is usually made by specialists (i.e., neurologists, psychiatrists, or gerontologists) and sporadically by a general practitioner (GP). However, only private health insurances cover such specialized services. In contrast, in many European countries most of patients with dementia are diagnosed by the GP and some patients are referred to neurologists or psychiatrists in private practice (Winblad et al., 2016). Both in LAC and in the EU only a very small proportion of patients are diagnosed in specialized centers such as memory clinics. Unlike EU [where the public health system tends to dominate (Winblad et al., 2016)], in most LAC the division of private and public health systems determines the quality and promptness of the diagnosis, as well as the proportion of people that can access health care facilities. At the public level, there are no centers of excellence providing multidisciplinary and individualized assessments. This, added to socioeconomic inequalities, emphasizes the importance of delineating actions toward these outstanding needs in LAC (Maestre, 2012).

In addition, basic recommendations and guidelines for dementia diagnosis are only available in some LAC (e.g., Chile, Argentina, and Brazil; Fuentes et al., 2008; Allegri, 2011; Caramelli et al., 2011; Chavez et al., 2011). In contrast, most of the EU countries have National Plans or guidelines for dementia diagnosis, the care for patients, and the recommended treatment (Winblad et al., 2016). Although some LAC has reached awareness regarding the importance of harmonizing diagnostic actions, this is not true for the regional level. The acceptance by scientific and academic communities about international guidelines on dementia is increasing, but with no adequate support from Latin American governments.

Regarding the diagnostic procedures, in most LAC, diagnosis of dementia is primarily clinical, and detailed cognitive assessments are offered mainly in private institutions. Diagnosis relies on the history, interview with the patient and the family, cognitive screening tests, and laboratory tests. Imaging and biomarkers are very restricted to a few private centers. In EU countries, the instruments employed for dementia diagnosis include comprehensive and detailed cognitive batteries, scales of functional impairment, informant-based questionnaires about basic and instrumental activities of daily living, and assessments of neuropsychiatric symptoms, quality of life, and disease burden. Structural neuroimaging is well established in the clinical diagnosis and the use of biomarkers is becoming part of the clinical routine in memory clinics (Winblad et al., 2016). Currently, dementia biomarkers are not sufficiently standardized for the use in everyday clinical practice, but standardization initiatives are ongoing in the EU countries. This kind of initiatives are lacking in LAC.

Finally, several cultural issues affect dementia diagnosis in LAC. For instance, low education and illiteracy are key problems affecting most LAC (Prince et al., 2003). The illiteracy rate in the older population is approximately 10% (Nitrini et al., 2009). This problem is highly relevant since the prevalence of dementia in illiterates is two times higher than that in literates (Nitrini et al., 2009). In addition, LAC are not homogenous in terms of language (e.g., aboriginal groups). However, neuropsychological tests used as part of the diagnosis process have been adapted and translated from those designed to assess populations with a different cultural background (Nitrini et al., 2004; Parra, 2014). The basis of cultural effects is poorly understood, but population-based studies (Sosa et al., 2009) suggest that normative data should not be generalized across populations with different sociocultural contexts. The extent to which these cultural factors are influencing the dementia prevalence in LAC needs to be investigated with upmost priority.

The key issues presented here highlight the importance of developing harmonized global strategies in LAC in order to overcome the existing barriers preventing an accurate and early dementia diagnosis. Some research groups in Latin America are already addressing relevant clinical issues regarding Alzheimer's disease (Parra et al., 2009, 2010, 2015; Pietto et al., 2016) and other dementias (Cardona et al., 2013; Ibáñez et al., 2013; Baez et al., 2014a,b, 2015, 2016a,b,c; Kargieman et al., 2014; Garcia-Cordero et al., 2015, 2016; Melloni et al., 2015, 2016; Santamaria-Garcia et al., 2016; Sedeno et al., 2016). The urgent need now is to develop and implement health-care strategies and national plans that meet the needs of individuals with dementia and their families. Though some LAC are developing national plans, important challenges remain to improve the quality of dementia diagnosis (Manes, 2016). Latin American governments should strengthen health services, improve training for health professionals to diagnose/treat dementia and promote the creation of public memory clinics. In facing the fight against dementia, LAC should capitalize on the experience of EU countries.

## Author contributions

Both authors developed the study concept, drafted the manuscript and approved its final version.

## Funding

This work was partially supported by grants from CONICET, CONICYT/FONDECYT Regular (1130920 and 1140114), FONCyT-PICT 2012-0412, FONCyT-PICT 2012-1309, FONDAP 15150012, and the INECO Foundation.

### Conflict of interest statement

The authors declare that the research was conducted in the absence of any commercial or financial relationships that could be construed as a potential conflict of interest.
